# Tissue eosinophilia: a morphologic marker for assessing stromal invasion in laryngeal squamous neoplasms

**DOI:** 10.1186/1472-6890-5-1

**Published:** 2005-01-07

**Authors:** Mahmoud Said, Sam Wiseman, Jun Yang, Sadir Alrawi, Wade Douglas, Richard Cheney, Wesley Hicks, Nestor Rigual, Thom Loree, Gregory Spiegel, Dongfeng Tan

**Affiliations:** 1 Department of Pathology, AmeriPath, Orlando, USA; 2 Department of Surgery St. Paul's Hospital/University of British Columbia, Vancouver, BC, Canada; 3 Department of Cancer Prevention, Roswell Park Cancer Institute, Buffalo, USA; 4 Department of Surgery, Roswell Park Cancer Institute, Buffalo, USA; 5 Department of Pathology, Roswell Park Cancer Institute, Buffalo, USA; 6 Department of Histopathology, King's College Hospital, Denmark Hill, London SE5 9RS, UK; 7 Department of Pathology and Laboratory Medicine, University of Texas Health Centre at Houston, 6431 Fannin, MSB 2.222, Houston, TX 77030, USA

## Abstract

**Background:**

The assessment of tumor invasion of underlying benign stroma in neoplastic squamous proliferation of the larynx may pose a diagnostic challenge, particularly in small biopsy specimens that are frequently tangentially sectioned. We studied whether thresholds of an eosinophilic response to laryngeal squamous neoplasms provides an adjunctive histologic criterion for determining the presence of invasion.

**Methods:**

Eighty-seven(n = 87) cases of invasive squamous cell carcinoma and preinvasive squamous neoplasia were evaluated. In each case, the number of eosinophils per high power field(eosinophils/hpf), and per 10 hpf in the tissue adjacent to the neoplastic epithelium, were counted and tabulated. For statistical purposes, the elevated eosinophils were defined and categorized as: focally and moderately elevated (5–9 eos/hpf), focally and markedly increased(>10/hpf), diffusely and moderately elevated(5–19 eos/10hpf), and diffusely and markedly increased (>20/10hpf).

**Results:**

In the invasive carcinoma, eosinophil counts were elevated focally and /or diffusely, more frequently seen than in non-invasive neoplastic lesions. The increased eosinophil counts, specifically >10hpf, and >20/10hpf, were all statistically significantly associated with stromal invasion. Greater than 10 eosinophils/hpf and/or >20 eosinophils/10hpf had highest predictive power, with a sensitivity, specificity and positive predictive value of 82%, 93%, 96% and 80%, 100% and 100%, respectively. Virtually, greater than 20 eosinophils/10 hpf was diagnostic for tumor invasion in our series.

**Conclusion:**

Our study suggests for the first time that the elevated eosinophil count in squamous neoplasia of the larynx is a morphologic feature associated with tumor invasion. When the number of infiltrating eosinophils exceeds 10/hpf and or >20/10 hpf in a laryngeal biopsy with squamous neoplasia, it represents an indicator for the possibility of tumor invasion. Similarly, the presence of eosinophils meeting these thresholds in an excisional specimen should prompt a thorough evaluation for invasiveness, when evidence of invasion is absent, or when invasion is suspected by conventional criteria in the initial sections.

## Background

Invasive squamous cell carcinoma(SC) is the most common malignancy of the larynx[1]. Distinguishing between preinvasive squamous neoplasia (high grade squamous cell dysplaisa/ squamous cell carcinoma in-situ, SCIS) and SC may be difficult in small biopsy specimens, particularly when the tissue is superficial and fragmented, a prominent inflammatory infiltrate obscures the epithelial-stromal interface, and/or there is tangential sectioning of the acanthotic neoplastic squamous epithelium. Even in larger resection specimens, the presence of invasion may sometimes be elusive if the invasive element lacks paradoxical maturation characterized by prominent eosinophilic cytoplasm that may undergo either central or individual cell keratinzation, well developed cell borders, and large vesicular nuclei with prominent nucleoli. The existence of an adjunctive feature associated with invasion would be helpful in assessing whether there is any degree of invasion in these challenging cases, or whether such a feature should raise the suspicion that the lesion may harbor an invasive component when it is absent by conventional diagnostic criteria.

A moderate to marked stromal eosinophils that may infiltrate into the neoplastic epithelium has occasionally been reported in invasive carcinoma [2-4]. Spiegel *et al *recently reported that the presence of eosinophils is associated with invasion in the neoplastic squamous lesions in the female genital tract, and proposed that eosinophilia provided as adjunctive morphologic feature in identifying SC in the cervix and vulva[5,6]. One of us (DT) has observed moderate to marked stromal eosinophilia in cases of SC of the larynx, whereas stromal eosinophils were usually either absent or rare in cases of laryngeal SCIS. We speculated that the degree of stromal eosinophilia is a pathologic feature that would provide an adjunctive criterion for distinguishing SC from SCIS in the larynx, and undertook a systematic study to test this hypothesis. In this study, we focused on a single head and neck region, the larynx, to avoid any potential selection bias, since squamous neoplasia and the associated host response and changes in the head and neck are heterogeneous and varied in different anatomic locations[7].

## Methods

The biopsy and resection specimens with available H&E stained slides of laryngeal SC and SCIS diagnosed at Roswell Park Cancer Institute from 1993 through 2000 were reviewed by two of the authors simultaneously (MZ and DT). Cases with prior radiation and/ or chemotherapy were excluded. All histology specimens at Roswell Park Cancer Institute were fixed in 10% formalin. Paraffin blocks of 5 μm thickness were cut and the sections were stained with conventional hematoxylin and eosin. For each biopsy and resection specimen, the original diagnosis was recorded and compared with the review diagnosis. Cases with any degree of invasion including "minimal invasion" or "microscopic invasion" in either a biopsy or resection specimen or both were classified as SC. Cases with SCIS only in a biopsy specimen that subsequently had invasion in the resection specimen were classified as SC. On the other hand, a resection specimen lacking invasion was required for a case to be classified as SCIS. For each of specimen, the high-power field(hpf) (Olympus BH2 ×10 ocular and ×40 objective lens) with a maximum number of eosinophils was identified and recorded as eos/hpf. Then, the eosinophils in the adjacent nine contiguous hpf were counted, added to those in the first, and recorded as eos/10hpf. Only nucleated cells with intensely red cytoplasmic granules were accepted as eosinophils, and care was taken to exclude red blood cells with superimposed mononuclear and polymorphonuclear inflammatory cells. And those that were confined to lymphovascular spaces were excluded. During the course of the study, it was noted that frozen section preparation results in the degranulation of eosinophils and leads to difficulty in their being recognized; however, under these conditions, collections extracytoplasmic typical red granules approximately the expected size of an eosinophil allows for their identification. As an internal control, non-neoplastic portions of the specimens, whenever available, were also evaluated.

### Statistical methods

Frequency was computed for each eosinophil category in invasive and non-invasive squamous neoplasia specimens obtained from biopsy and excision. Chi-square test was utilized to examine the difference of frequency distribution between each elevated eosinophil category and the referent eosinophil category (0–4 eos/10hpf). This analysis was conducted independently for specimens obtained from biopsy and excision. Sensitivity, specificity, positive predictive value and negative predictive value were computed for eosinophil counts that were exceeding 10 eos/hpf or 20 eos/10hpf to evaluate if these two classifications of eosinophil count would suggest any significance of clinical implication from statistical point of view. P value less than 0.05 was used to determine statistical significance. Elevated eosinophils were defined and categorized as: focally and moderately elevated (5–9 eos/hpf), focally and markedly increased(>10/hpf), diffusely and moderately elevated(5–19 eos/10hpf), and diffusely and markedly increased (>20/10hpf), while 0–4 eosinophils/10hpf was used as a baseline. The recorded eosinophil counts were analyzed to determine whether there were thresholds of stromal versus neoplastic squamous eosinophils per 1 hpf and 10 hpf that were significantly associated with invasive tumor in both biopsy and follow-up excisional/resectional specimens.

## Results

A total of 87 cases were evaluated and sixty-eight percent of the cases (n = 59) displayed a chronic inflammation in the stroma. Fifty-seven were biopsy specimens and 30 were ablative resection specimens. The diagnoses of 57 biopsy specimens were 35 invasive carcinoma, 4 minimal invasive carcinoma, 2 suspicious for invasion, and 16 were preinvasive squamous cell neoplasia. 27 of the biopsy specimens (18 invasive carcinoma, 1 minimal invasive carcinoma, 1 suspicious for invasion and six preinvasive squamous cell neoplasm) were followed by ablative resections (7 wide excisional resection, and 20 total laryngectomy). The follow-up specimens confirmed 19 invasive carcinoma and six preinvasive carcinoma, and revealed an invasive carcinoma in the suspicious case. In this case, there were stromal nests of neoplastic squamous cells that were associated with 15 and 34 eosinophils per 1 and 10 hpf, respectively, and clinical image studied revealed an advanced disease (stage IIIa). Additionally, Other 3 ablative resection specimens (all excisional biopsies) were initial operations. There were 2 invasive carcinoma and 1 preinvasive lesion.

**Figure 1:**
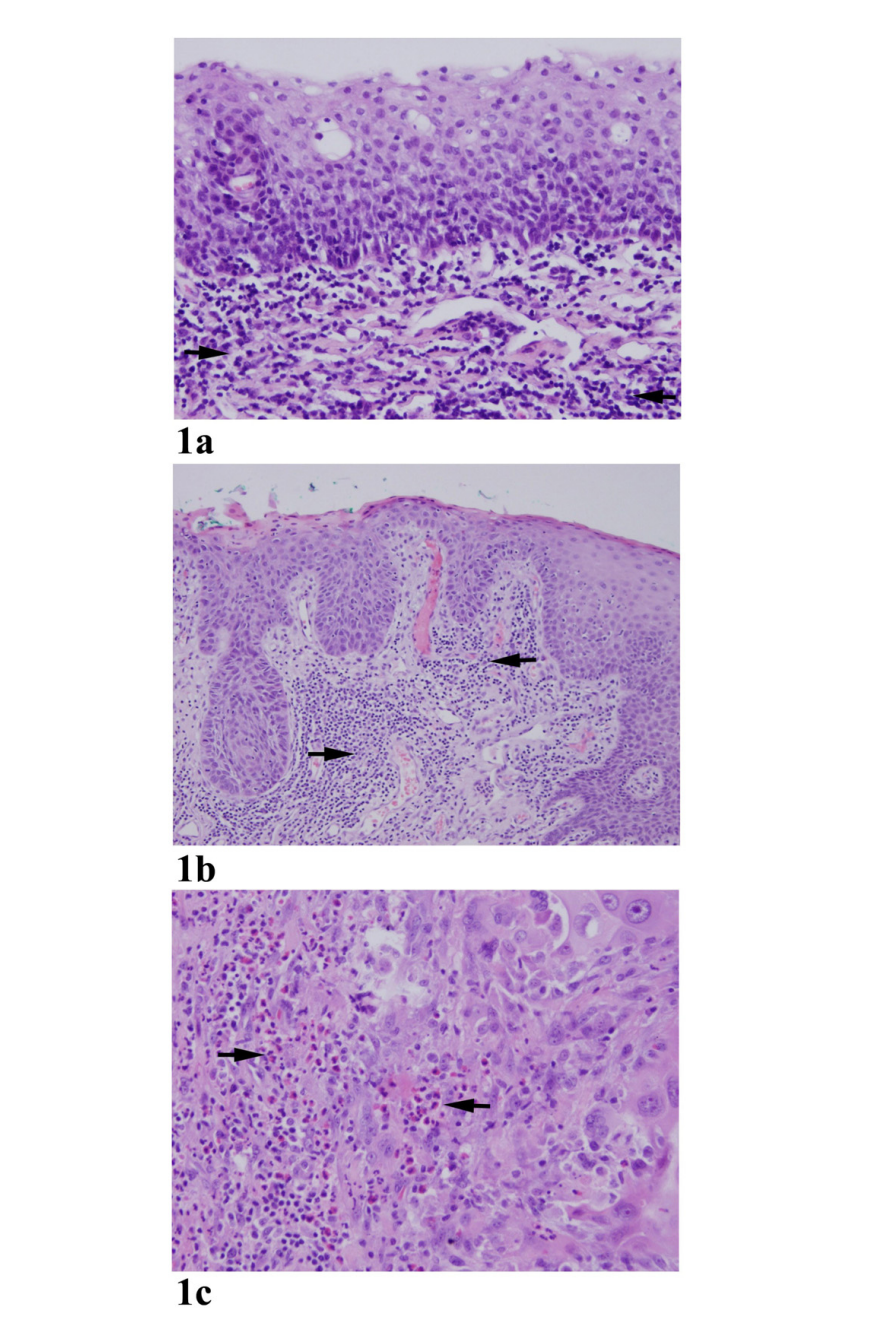
a. Absence of eosinophils in normal squamous epithelium. Note that a moderately inflamed submucosal tissue. Arrows point to the inflammatory cells. 200× b. A squamous cell carcinoma in-situ (non-invasive tumor) with no elevated eosinophils in a chronic inflammatory background. Arrows point to the inflammatory cells. 200× c. Markedly increased eosinophils in an invasive squamous cell carcinoma. Note that the eosinophils (arrows) were a major component of the infiltrating nucleated cells. 200×

The distribution of eosinophil counts is summarized in Table 1. Both diffuse and focal elevated eosinophilic infiltration were noted in invasive tumor and, to much less extend, in the non-invasive counterparts. Typical examples of eosinophilic infiltration in the stroma tissue were illustrated in Figures 1A–1C. As shown in Table 2, thirty-six (57%) of patients with invasive squamous cell carcinoma were found to have diffuse eosinophilic infiltration (>20/10hpf), whereas elevated eosinophils were not diffusely observed in all non-invasive lesions (p < 0.05). The same held true for focally marked eosinophilia (>10/hpf) when compared invasive group with non-invasive group. One exception was observed in a non-invasive neoplasia (Case 37, high grade dysplasia/SCIS) which showed a marked increased eosinophils (10 eosinophils/hpf). Noticeably, this high grade dysplasia with elevated eosinophil infiltrate uncovered an invasive carcinoma in the follow-up resection specimen. These results indicated that a close association exists between the stromal invasion and the presence of elevated tissue eosinophils. Stromal eosinophilia was statistically significantly associated with invasion in squamous cell carcinoma (Table 2).

**Table 1:** Distribution of eosinophils in invasive and non-invasive squamous neoplasia

Specimen and Diagnosis	Eosinophils/hpf					Eosinophils/10hpf			
	0	1–4	5–9	10–20	>20	0	1–4	5–19	20 or greater
		
Biopsy									
Invasive CA(41)	1(2%)	5(12%)	8(20%)	15(36%)	12(29%)	1(3%)	5(12%)^1^	10(24%)^1^	25(61%)^1^
Non-invasive(16)	13(81%)	1(6%)	1(6%)	1(6%)	none	7(44%)^2^	6(38%)^2^	3(18%)^2^	none
Excision/Resection									
Invasive CA(22)	1(5%)	4(18%)	8(36%)	6(23%)	3(14%)	1(5%)	3(14%)	7(31%)	11(50%)
Non-invasive(8)	6(76)	1(12%)	1(12%)	none	none	4(50%)	3(38%)	1(12%)	none

^1^Two invasive carcinoma case less than 10hpf in size

^2^One non-invasive carcinoma case less than 10hpf in size.

**Table 2:** Significance of eosinophils in invasive and non-invasive squamous neoplasia

Lesion	Eosinophils Counts							
	5–9 eos/hpf ^1^	p	>or = 10 eos/hpf	p	5–19 eosin/10hpf^2^	p	>or = 20 eos10/hpf	p
	
Invasive CA in biopsy	8/41(20%)	>0.05	27/41(66%)	<0.01	10/41(24%)	>0.05	25(61%)	<0.005
Non invasive lesion in biopsy	1/16(6%)		1/16(6%)		3/16(18%))		0/16(--)	
Invasive CA in Excision	8/22(36%)	>0.05	9/22(41%)	<0.05	7(31%)	>0.05	11((50%)	<0.05
Non-invasive lesion in excision	1/8(12%)		0/8(--)		1(12%)		0/8(----)	

^1 ^*eos/hpf: *for each specimen, a high power field (hpf) (Olympus BH2 ×10 ocular and ×40 objective lens) with a maximum number of eosinophils in the lesion area

^2 ^*eos/10hpf*: for each specimen, one high power field (hpf) (Olympus BH2 ×10 ocular and ×40 objective lens) with a maximum number of eosinophils in the lesion area and additional nine hpfs in the contiguous areas

**Figure 2:**
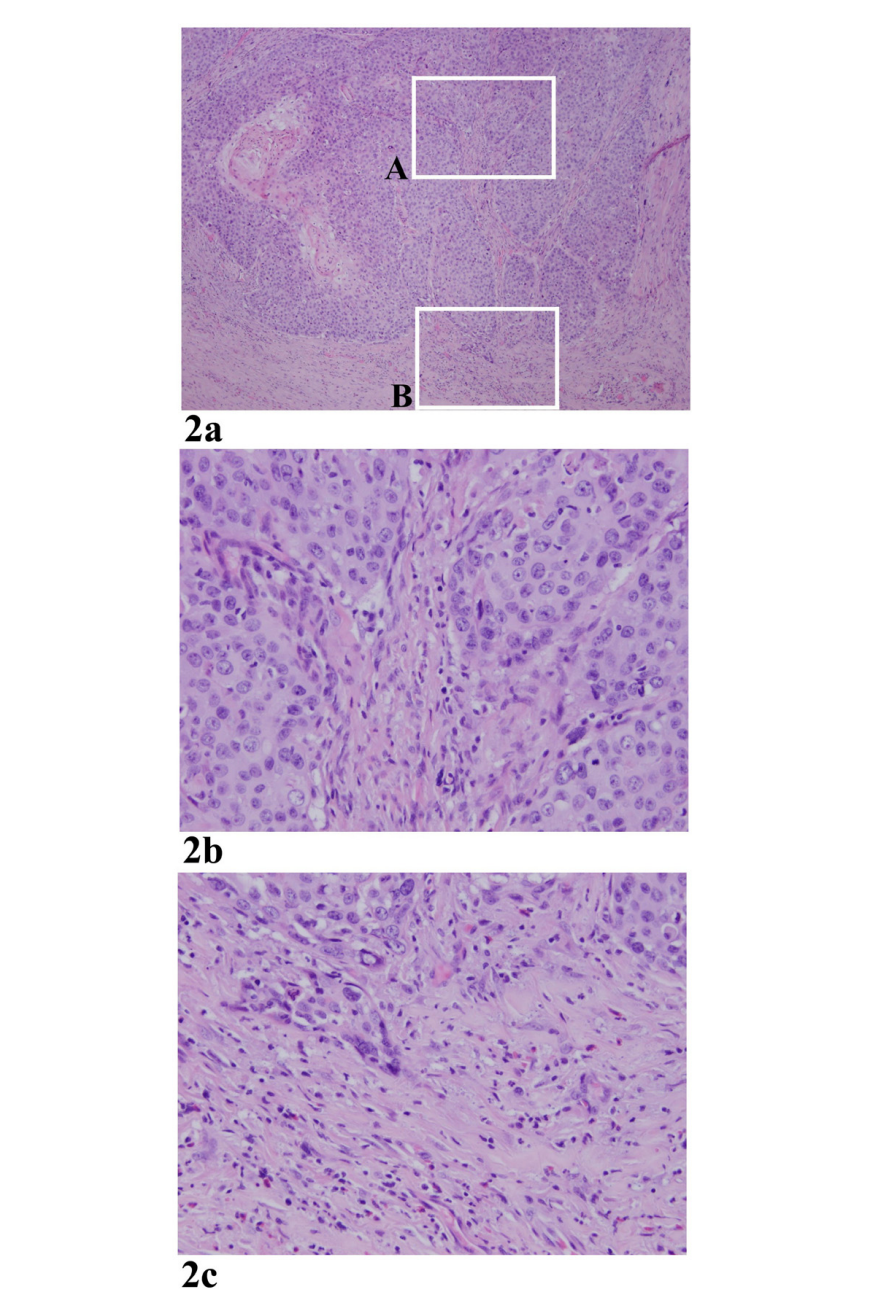
a. A low power view of an invasive carcinoma. Note that there was no significant inflammatory background. 40× b. A higher power view of the square area labeled as A in Figure 2a. No eosinophils were present in the stromal tissue between the tumor nests. 200× c. A higher power view of the square area labeled as B in Figure 2a. Elevated eosinophils were present at the invading front of the carcinoma. 200×

There is no association of elevated tissue eosinophils with overall inflammatory response of the stroma in the specimens studied (p < 0.05). Specifically, among the 37 cases with > = 10 eosinophils/hpf, 24 cases displayed a non-specific inflammation, while 50 cases with <10 eosinophils/hpf, 35 displayed a non-specific inflammation. Among the 36 cases with > = 20 eosinophils/10hpf, 24 cases revealed a non-specific inflammation, while 51 cases with <20 eosinophils/10hpf, 34 revealed a non-specific inflammation. In fact, some invasive carcinomas (n = 6) virtually contained no chronic inflammatory background, but showed a marked elevated tissue eosinophila (Fig. 2). In addition, a number of cases (n = 21) with elevated eosinophila showed a distinct polarization of the infiltrating cells, namely eosinophilic cells accumulating in the tumor invading front (Fig. 2C).

The predictive values of tissue eosinophils in assessing stromal invasion in squamous neoplastic lesions of larynx are presented in Table 3. In biopsy specimens, diffusely elevation of eosinophils (>20/10 hpf) had a sensitivity, specificity and positive predictive value of invasion of 80%, 100% and 100%, respectively. In these specimen, the presence of >10 eosinophils/hpf predicted invasion in all cases with a sensitivity of 81% and positive predictive value 96%, respectively, while values below this threshold had a predictive value of an absence of invasion 68%. Similarly, the presence of > 20 eosinophils/10hpf in the excisional specimens had a sensitivity, specificity, and positive predictive value of invasion of 69%, 100% and 100 %, respectively. In these excisional specimens, the presence of >10 eosinophils/hpf had sensitivity, specificity, and positive predictive values for invasion of 64%, 100% and 100%, respectively. Values below the thresholds of >10 eosinophils/hpf or 20 eosinophils/10hpf had a predictive value of an absence of invasion of 40% and 42%, respectively.

**Table 3:** Predictive value of eosinophils in assessing stromal invasive in squamous neoplasia of larynx

Eosinophils in lesion	Sensitivity(%)	Specificity(%)	Positive predictive value(%)	Negative predictive value(%)
Biopsy specimens				
>or = 10 eos/hpf	66%	94%	96%	52%
>or = 20/10hpf	80%	100%	100%	68%
Excisonal specimens				
>or = 10 eos/hpf	64%	98%	100%	58%
>or = 20 eos/10 hpf	68%	100%	100%	58%

Sensitivity, specificity, positive predictive value and negative predictive value were computed for eosinophil counts that were exceeding 10 eos/hpf or 20 eos/10hpf to evaluate if these two classifications of eosinophil count would suggest any significance of clinical implication from statistical point of view

Sections with adequate non neoplastic epithelium were available in twelve cases. Eight of them were absent for eosinophils. The highest counts for non-neoplastic epithelium were 4 eosinophils/hpf and 8 eosinophils/10hpf. Although it seems that non-neoplastic epithelium contains less eosinophils than squamous neoplasia, no statistical analyses were performed since the number of available non-neoplastic regions in this series was too small.

## Discussion

For decades, pathologists have used a variety of histologic features, including desmoplastic stromal reaction, intrastromal foreign body reaction to keratin, and the presence of separate minute clusters of intrastromal neoplastic cells, to assess and identify invasion[8,9]. However, when evaluating a small, poorly-oriented, tangentially-cut specimens, one sometimes enters an area replete with uncertainties. The presence of a morphologic feature associated with invasion would be helpful in determining whether any degree of invasion has occurred in the equivocal cases. In practice, we have noticed a frequent presence of eosinophilic infiltration in invasive squamous cell carcinoma of the larynx, which is usually absent in non-invasive neoplastic counterparts. Such a consistent observation has prompted us to carry out the current study.

In this series, a systematic study of eosinophils in tissues of squamous neoplasia of larynx suggests that elevated eosinophils are a morphologic marker for assessing tumor invasiveness. We observed that in the invasive squamous carcinomas eosinophils were significantly elevated focally and /or diffusely, statistically more frequent than in non-invasive neoplasia. The increased eosinophil counts (>10 hpf, and >20/10 hpf) in laryngeal biopsy and excisional specimens were all statistically significantly associated with stromal invasion. In contrast, values below both of these thresholds had a significant predictive value for the absence of invasion. The slight decrease in the correlation of >10 eosinophils/hpf with invasion in excisional specimens, relative to that in biopsy counterparts, may be attributed to the increased chance of observing microscopic clusters of eosinophils unrelated to invasion in the larger specimens.

It is not surprising to observe inflammation in the specimens examined, likely due to several factors including the specific anatomic location and an overall inflammatory response of the stroma to the tumor, among others[7,9]. However, there is no association of elevated eosinophils with overall inflammatory response of the stroma in the specimens studied. Furthermore, a number of cases with elevated eosinophila showed a distinct polarization of the infiltrating cells, specifically eosinophilic cells accumulating in the tumor invading front (Fig. 2C). Cumulatively, our findings strongly indicate that elevated tissue eosinophila is a specific cell response independent of a non-specific inflammatory reaction.

Although elevated eosinophil counts are statistically significantlly associated with stromal invasion in squamous cell carcinoma of larynx, occasionally the presence of high number of eosinophils were observed in the non-invasive counterpart tissues (Table 1). In other words, the presence of eosinophils in squamous neoplasia of larynx is not pathognomonic for stromal invasion and caution must be exerted when evaluating the number of infiltrating eosinophils. However, the quantitation method and thresholds identified in the current experiment may represent an adjunctive feature in assessment of stromal invasion in squamous neoplasia. Specifically, the presence of eosinophils at these thresholds should raise the suspicion that invasive or microinvasive carcinoma is present within the specimen, particularly when >10 eosinophils/hpf and or 20 eosinophils/10hpf are observed.

Since the first observation of malignancy with marked blood eosinophilia described by Rheinbach in 1893, eosinophilia has been described in human cancers from a variety of organs [10-12]. In head and neck squamous cell carcinoma, it has been reported that the presence of tissue eosinophils ranges between 22 and 89% [13-16]. Most of these series have focused on whether the presence of a prominent eosinophilic infiltrate has a prognostic value, or is an indicator of response to treatment. Some authors have claimed that the presence of a marked or moderate eosinophilic is associated with a poor prognosis[12,17], while others have found that eosinophilia is a favorable prognostic feature[13,14]. No study has addressed the value of eosinophils in distinguishing invasive from non-invasive squamous neoplasia in the head and neck.

The mechanism of eosinophilic accumulation in cases of invasive carcinoma remains largely unknown. It has been suggested that such eosinophilic infiltration may be induced by a tumor-derived eosinophil chemotactic factor[18,19]. A recent study further indicated that stromal eosinophils in squamous cell carcinoma may play a key role in tumor invasion through activation of gelatinase[20,21]. It was found that 92-kd gelatinase, a key member of the matrix metalloproteineaes which are involved in tumor invasion by breaking down the basement membrane and extracellular matrix, is actively expressed by eosinophils.

In conclusion, although the etiology of tissue eosinophils in invasive carcinoma is unknown, our study is the first to suggest that an elevated eosinophil count in the squamous neoplasia of larynx may serve as a morphologic feature associated with tumor invasion. The presence of more than individual eosinophils, specifically when the number of infiltrating eosinophils exceeds 10/hpf and or >20/10 hpf in a biopsy of larynx with squamous neoplasia, represents a histologic marker for the presence of tumor invasion. Similarly, the presence of eosinophils reaching these thresholds in an excisional specimen should prompt a thorough search for invasiveness when evidence of invasion is absent, or when invasion is suspected by conventional criteria in the initial sections. Although the present study assesses a quantitative parameter of tumor invasion, in our daily practice we find it useful that a readily appreciable elevation of tissue eosinophilia alerts us to search for possible invasiveness in tissue biopsy of laryngeal lesions.

## Competing interests

The author(s) declare that they have no competing interests.

## Authors' contributions

Drs. Said, Speiegel, Tan for study design; Drs. Said, Tan, for pathology evaluation; Drs. Alrwi, Douglas, Hicks, Loree, Riguel, Wiseman for surgical evaluation and clinical follow-up as well as card review. Dr. Yang for statistical analyses. Dr. Cheney for administrative and financial support.

## Pre-publication history

The pre-publication history for this paper can be accessed here:


